# Integrated Bioinformatics Analysis Reveals Key Candidate Genes and Pathways Associated With Clinical Outcome in Hepatocellular Carcinoma

**DOI:** 10.3389/fgene.2020.00814

**Published:** 2020-07-24

**Authors:** Yubin Li, Runzhe Chen, Jian Yang, Shaowei Mo, Kelly Quek, Chung H. Kok, Xiang-Dong Cheng, Saisai Tian, Weidong Zhang, Jiang-Jiang Qin

**Affiliations:** ^1^School of Pharmacy, Naval Medical University, Shanghai, China; ^2^Department of Thoracic/Head and Neck Medical Oncology, The University of Texas MD Anderson Cancer Center, Houston, TX, United States; ^3^Department of Genomic Medicine, The University of Texas MD Anderson Cancer Center, Houston, TX, United States; ^4^The First Affiliated Hospital of Zhejiang Chinese Medical University, Hangzhou, China; ^5^Accenture Applied Intelligence, ASEAN, Singapore, Singapore; ^6^Precision Medicine Theme, South Australian Health and Medical Research Institute, Adelaide, SA, Australia; ^7^Discipline of Medicine, Adelaide Medical School, The University of Adelaide, Adelaide, SA, Australia; ^8^Institute of Cancer and Basic Medicine, Chinese Academy of Sciences, Hangzhou, China; ^9^Cancer Hospital of the University of Chinese Academy of Sciences, Hangzhou, China; ^10^Zhejiang Cancer Hospital, Hangzhou, China

**Keywords:** hepatocellular carcinoma, differentially expression genes, enrichment analysis, survival analysis, prognosis

## Abstract

Hepatocellular carcinoma (HCC) accounts for approximately 85–90% of all liver cancer cases and has poor relapse-free survival. There are many gene expression studies that have been performed to elucidate the genetic landscape and driver pathways leading to HCC. However, existing studies have been limited by the sample size and thus the pathogenesis of HCC is still unclear. In this study, we performed an integrated characterization using four independent datasets including 320 HCC samples and 270 normal liver tissues to identify the candidate genes and pathways in the progression of HCC. A total of 89 consistent differentially expression genes (DEGs) were identified. Gene-set enrichment analysis revealed that these genes were significantly enriched for cellular response to zinc ion in biological process group, collagen trimer in the cellular component group, extracellular matrix (ECM) structural constituent conferring tensile strength in the molecular function group, protein digestion and absorption, mineral absorption and ECM-receptor interaction. Network system biology based on the protein–protein interaction (PPI) network was also performed to identify the most connected and important genes based on our DEGs. The top five hub genes including osteopontin (*SPP1*), Collagen alpha-2(I) chain (*COL1A2*), Insulin-like growth factor I (*IGF1*), lipoprotein A (*LPA*), and Galectin-3 (*LGALS3*) were identified. Western blot and immunohistochemistry analysis were employed to verify the differential protein expression of hub genes in HCC patients. More importantly, we identified that these five hub genes were significantly associated with poor disease-free survival and overall survival. In summary, we have identified a potential clinical significance of these genes as prognostic biomarkers for HCC patients who would benefit from experimental approaches to obtain optimal outcome.

## Introduction

Liver cancer is the fourth leading cause of cancer-related death worldwide and ranks sixth in terms of incidence (Bray et al., 2018; Villanueva, 2019). Among all types of primary malignant liver tumors, hepatocellular carcinoma (HCC) accounts for approximately 85–90% of all cases. The major risk factors including chronic infections by hepatitis B virus (HBV) and hepatitis C virus (HCV), aflatoxin exposure, smoking, type 2 diabetes, obesity, and so on (Marengo et al., 2016; Bray et al., 2018; Phukan et al., 2018). As a highly heterogeneous cancer disease, localized HCC patients often have poor prognosis with 5-year overall survival (OS) rate of 30%, and this rate drops below 5% for those with distant metastases (Oweira et al., 2017). For patients at early disease stages, liver resection is the most effective treatment option, however, only less than 30% of HCC patients are eligible surgery, and among those around 70% eventually relapse within 5 years after treatment (Waghray et al., 2015). Over the past few decades, despite advances in chemotherapy, targeted therapy, radiation therapy, and immunotherapy in the clinical arena, the survival of HCC patients has not significantly increased, and translational studies to understand the mechanisms and prognosis remain underwhelming to design novel therapeutic strategies (Visvader, 2011; Aravalli et al., 2013; Llovet et al., 2018).

Data, information, knowledge and wisdom (DIKW) model has been widely used in life in all aspects including medicine (Song et al., 2018, 2020; Duan, 2019a, b; Duan et al., 2019a, b). In recent years, genome-wide profiling has substantially advanced our understanding of the genetic landscape and driver pathways leading to HCC (Totoki et al., 2014; Schulze et al., 2015; Zucman-Rossi et al., 2015; Ally et al., 2017; Villanueva, 2019), revealing Cellular tumor antigen p53 (*TP53*), Catenin beta-1 (*CTNNB1*), Axin-1 (*AXIN1*), Telomerase reverse transcriptase (*TERT*) promoter and other key genes as driver mutations, and WNT/β-catenin, p53 cell cycle pathway, oxidative stress, PI3K/AKT/MTOR, and RAS/RAF/MAPK pathways as key signaling pathways involved in liver carcinogenesis. However, existing studies have been of limited sample size that failed to create molecular prognostic indices and also the inconsistent computational methods may have restricted the power to identify potential meaningful molecular biomarkers and new therapeutic targets. Therefore, an integrated bioinformatics study combining the most updated genomic data thus providing novel insight into the mechanisms underlying therapeutic resistance and disease progression is highly warranted.

Microarray technology has become an indispensable tool to monitor genome wide expression levels of genes in a given organism and has been successfully used to classify different types of cancer and predict clinical outcomes (Trevino et al., 2007). These microarray technologies have also been applied in many studies to define global gene expression patterns in primary human HCC in an attempt to gain insight into the mechanisms of hepatocarcinogenesis (Crawley and Furge, 2002; Woo et al., 2008; Hoshida et al., 2009; Villanueva et al., 2012; Jin et al., 2015). In the present study, we selected four independent datasets consisting a total of 320 HCC cases and 270 cases of normal liver tissues in the Gene Expression Omnibus (GEO) database to identify reliable markers and pathway alterations linked with the pathogenesis of HCC cases (Wurmbach et al., 2007; Mas et al., 2009; Roessler et al., 2010). We identified 89 differential expression genes (DEGs) including 31 up-regulated genes and 58 down-regulated genes. Gene ontology (GO) analysis revealed cellular response to zinc ion in biological process (BP) group, collagen trimer in the cellular component (CC) group, and extracellular matrix (ECM) structural constituent conferring tensile strength in the molecular function (MF) group. Further pathway enrichment analysis revealed that enrichment in protein digestion and absorption, mineral absorption, propanoate metabolism, and ECM-receptor interaction. Finally, the top five hub genes osteopontin (*SPP1*), Collagen alpha-2(I) chain (*COL1A2*), Insulin-like growth factor I (*IGF1*), lipoprotein A (*LPA*), and Galectin-3 (*LGALS3*) were identified from the protein–protein interaction (PPI) network and those highly altered genes were validated by western blot assay and Immunohistochemistry (IHC) analysis and found to be associated with clinical outcome of HCC patients.

## Materials and Methods

### Data Source and Identification of DEGs

Microarrays data were obtained from the Oncomine 4.5 database^1^ contains 715 datasets and 86,733 samples. Of which, we filtered four datasets comprising Mas liver (GSE14323, containing 19 liver tissues and 38 HCCs), Roessler liver (GSE14520 based on GPL571 platform, containing 21 liver tissues and 22 HCCs), Roessler liver 2 (GSE14520 based on GPL3921 platform, containing 220 liver tissues and 225 HCCs), and Wurmbach liver (GSE6764, containing 10 liver tissues and 35 HCCs) after using the following criteria: (a) Analysis type: cancer vs. normal analysis; (b) Cancer type: hepatocellular carcinoma; (c) Data type: mRNA; (d) Sample type: clinical specimen; (e) Microarray platform: Human Genome U133A, U133A 2.0, or U133 Plus 2.0. A total of 270 cases of normal liver tissues and 320 cases of HCCs were included in the integrated analysis. To analyze the DEGs between HCC and normal liver tissues, the data were then processed on GEO2R website^2^. The differentially expressed genes were identified using limma R package at a cutoff | logFC| > 1 and adjusted *p* value < 0.05 (Benjamini & Hochberg).

### GO and Pathways Enrichment Analysis

The annotation function of GO analysis is comprised of three categories: BP, CC, and MF. Kyoto Encyclopedia of Genes and Genomes (KEGG) is a database resource for understanding high-level functions and utilities of the genes or proteins (Kanehisa and Goto, 2000; Kanehisa et al., 2012, 2016). GO analysis and KEGG pathway enrichment analysis of candidate DEGs were performed using the R package “clusterProfiler.” Reactome^3^ was also used for pathway enrichment analysis (Fabregat et al., 2018). Adjusted *p*-value less than 0.05 was considered as the cut-off criterion for both GO analysis and pathway enrichment analysis.

### PPI Network and Modular Analysis

Protein–protein interaction network was constructed to determine the importance of these DEGs by comparing the interactions between different DEGs. STRING database^4^ and Cytoscape software (3.7.2 version) were applied to construct and visualize the PPI networks (Szklarczyk et al., 2017), followed by Molecular Complex Detection (MCODE) plug-in in Cytoscape for selecting significant modules of hub genes from the PPI network (Bader and Hogue, 2003), with the following criteria: degree cutoff (number of connections with other nodes) ≥ 2, node score cutoff (the most influential parameter for cluster size) ≥ 2, K-core (This parameter filters out clusters that do not contain a maximally inter-connected sub-cluster of at least k degrees. For example, a triangle including three nodes and three edges is a two-core representing two connections per node. Two nodes with two edges between them meet the two-core rule as well) ≥ 2 and max depth (this parameter limits the distance from the seed node within which MCODE can search for cluster members) = 100. KEGG pathway enrichment analysis of the modules was carried out using the online DAVID database^5^ (Huang da et al., 2009).

### Hub Gene Selection and Prognostic Analysis

Hub genes were selected based on comparison of top 10 genes ranked by degree and betweenness centrality Network of hub genes. Their co-expressed genes were then analyzed using cBioPortal online platform^6^ (Gao et al., 2013). Genetic alterations of these hub genes were explored and compared using the cBioPortal database. Biological process analysis of hub genes was then performed and visualized using plug-in Biological Networks Gene Oncology tool (BiNGO) app in Cytoscape software (Maere et al., 2005). Stage-related information analysis based on gene expression was performed in UALCAN^7^, a comprehensive web resource for analyzing omics data (Chandrashekar et al., 2017). Disease-free survival (DFS) is a concept used to describe the period after a successful treatment of cancer. OS means the length of time from either the date of diagnosis or the start of treatment for HCC. DFS and OS are both measured to see how well a new treatment works. DFS and OS analysis associated with these hub genes were performed using the Kaplan–Meier Plotter online database^8^.

### Cell Lines and Cell Culture

Hepatocellular carcinoma cell lines Hep3B and HepG2 and human normal liver cell line L02 were obtained from Shanghai Institute of Biochemistry and Cell Biology, Chinese Academy of Sciences. All of these cell lines were cultured in Dulbecco’s modified Eagle’s medium (DMEM) (catalog number 10569010, Gibco) containing 10% (v/v) fetal bovine serum (FBS) (catalog number 10091148, Gibco) supplemented with 1% (v/v) penicillin streptomycin solution (catalog number SV30010, Hyclone) (containing 100 U/ml penicillin and 100 μg/ml streptomycin) in a humidified incubator at 37°C with 5% CO_2_.

### Protein Preparation and Western Blot Analysis

Briefly, HCC cells were lysed with cold M-PER lysate buffer (catalog number 78501, Roche) [containing 1 × protease inhibitors (catalog number 11836153001, Roche) and phosphatase inhibitor cocktail (catalog number 78420, Roche)] and centrifuged at 4°C for 10 min. The protein concentrations of collected supernatants were determined by the BCA protein assay kit (catalog number P0011, Beyotime). Equal amounts of total proteins were separated in 10 or 12% SDS-PAGE and transblotted onto the 0.45 μm PVDF membranes (catalog number 1620177, BIO-RAD). The membranes were blocked in 5% fat-free milk in TBST (150 mM NaCl, 50 mM Tris, pH 7.2) for 1 h at room temperature and subsequently incubated with corresponding primary antibodies as following: anti-SPP1 (catalog number ab8448, Abcam, 1:1000), anti-COL1A2 (catalog number 66761-1-lg, Proteintech, 1:1000), anti-IGF1 (catalog number ab9572, Abcam, 1:1000), anti-LGALS3 (catalog number ab209344, Abcam, 1:1000), anti-GADPH (catalog number 10494-1-AP, Proteintech, 1:10000), and anti-β-Tubulin (catalog number T0023, Affinity, 1:20000) at 4°C overnight, followed by incubation with a donkey anti-mouse (catalog number C61116-02, LI-COR) or goat anti-rabbit (catalog number C80118-05, LI-COR) secondary antibody for 1 h at room temperature. Then membranes were scanned using the Odyssey infrared imaging system (LI-COR) and the images were captured. The gray levels of the bands were determined by Image J software. The expression of proteins was normalized using the GADPH or β-Tubulin values. The assay was performed three independent times.

### Patient Samples

This study was approved by Cancer Hospital of the University of Chinese Academy of Sciences; Zhejiang Cancer Hospital. Twenty formalin-fixed, paraffin-embedded (FFPE) HCC tissues and corresponding adjacent non-cancerous tissues were collected from the Department of Abdominal Surgery, Zhejiang Cancer Hospital. All FFPE HCC tissues were screened by two pathologists independently to confirm the diagnosis of HCC. The most representative tumor and non-cancerous tissues were selected for immunohistochemistry analysis.

### IHC Analysis

Neutral 10% buffered formalin-fixed tissue specimens were embedded in paraffin wax and then sliced to 4-micron thick sections by a microtome. In brief, the tissue slices were firstly deparaffinized, followed by rehydration and a 10-min boiling in 10 mmol/L citrate buffer (pH = 6.4) for antigen retrieval. Then, the sections were treated in methanol containing 3% H_2_O_2_ for 20 min to inhibit the endogenous tissue peroxidase activity. After being blocked with 1% bovine serum albumin (BSA) at 37°C for 30 min, IHC staining was carried out for the protein expression of SPP1, COL1A2, IGF1, and LGALS3 using specific primary antibodies at 4°C overnight, followed by staining with species-specific secondary antibodies labeled with horseradish peroxidase (HRP). The slides were developed in diaminobenzidine (DAB) and counter-stained with hematoxylin. Then images of the sections were photographed using an Olympus microscope (Olympus Life Science). The clinical specimen data of LPA were obtained from The Human Protein Atlas database^9^.

### Statistical Analysis

Statistical analysis was performed using GraphPad Prism software (version 8.0.1) and R software (version 3.4.2^10^). *p*-value < 0.05 was considered statistically significant. The column diagram was graphed with GraphPad Prism software (version 8.0.1).

## Results

### Data Source and Analysis

**FIGURE 1:**
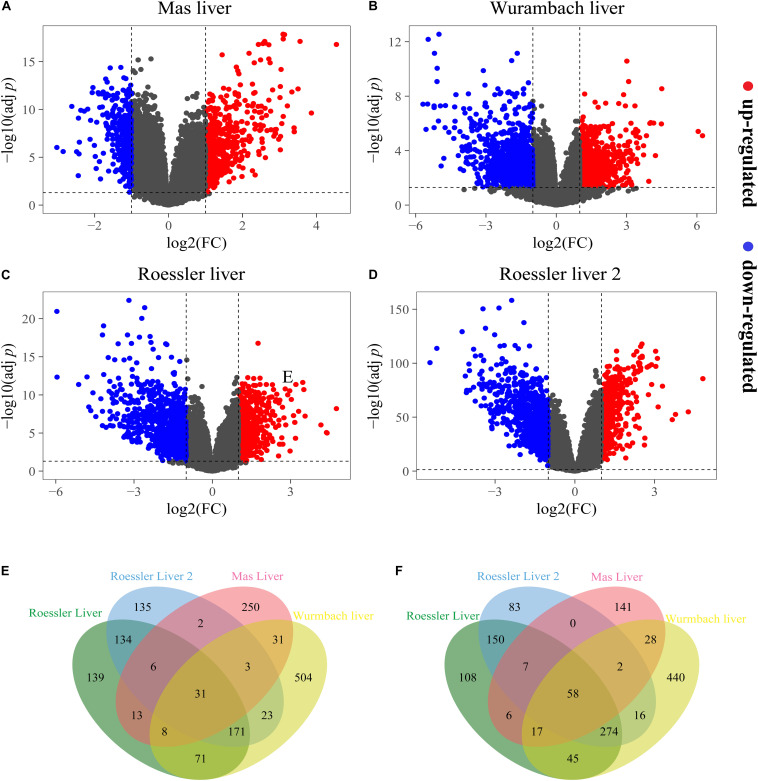
The DEGs screened from four independent datasets. Up-regulated DEGs (red-colored dots) and down-regulated (blue-colored dots) DEGs are selected with [logFC] > 1 and adjust *p*-value < 0.05 from the mRNA expression profiling sets **(A)** Mas liver, **(B)** Wurmbach liver, **(C)** Roessler liver, and **(D)** Roessler liver 2. Venn diagram showed **(E)** 31 consistently up-regulated DEGs and **(F)** 58 consistently down-regulated DEGs in four datasets.

A total of 603, 1,238, 1,095, and 1,722 DEGs have been extracted from the four independent expression datasets Mas liver, Roessler liver, Roessler liver 2, and Wurmbach liver, respectively (Figures 1A–D, Table 1, and Supplementary Tables S1, S2). The comparison of all genes from these datasets identified 89 consistently and significantly dysregulated genes, including 31 up-regulated genes and 58 down-regulated genes in HCC compared to normal liver tissues (Figures 1E,F and Supplementary Table S3). Notably, several genes such as Glypican-3 (*GPC3*) (Wurmbach et al., 2007) and *SPP1* (Roessler et al., 2010) have been reported previously, proving the feasibility of the method.

**TABLE 1:** Details of the four HCC datasets.

**Datasets**	**GSE**	**Tumor**	**Normal**	**References**
Mas liver	GSE14323	38	19	Mas et al., 2009
Roessler liver	GSE14520(GPL571 platform)	22	21	Roessler et al., 2010
Roessler liver 2	GSE14520(GPL3921 platform)	225	220	Roessler et al., 2010
Wurmbach liver	GSE6764	35	10	Wurmbach et al., 2007

*HCC, Hepatocellular carcinoma.*

### GO Analysis of DEGs in HCC

**FIGURE 2:**
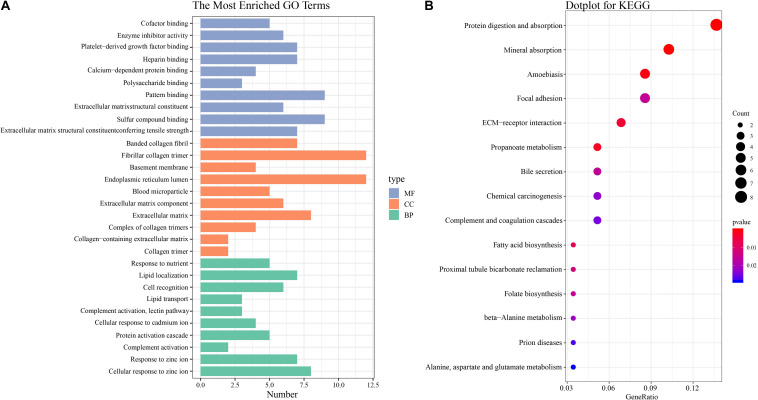
GO and KEGG pathway enrichment analysis **(A)** GO analysis of all DEGs. The most enriched GO terms are listed in the diagram. **(B)** KEGG pathway analysis of all DEGs. The most enriched KEGG pathways are shown in the picture.

The functional characteristics of these 89 DEGs were explored using GO analysis and were grouped into BP, cell component and MF (Figure 2A). Overall, cellular response to zinc ion covering five genes was found to be the dominant BP. Collagen trimer covering seven genes was found to be the top CC. ECM structural constituent conferring tensile strength covering five genes was the top MF. As shown in Table 2, in the BP group, up-regulated genes were mainly enriched in ECM organization, epithelial tube morphogenesis, and positive regulation of leukocyte migration while down-regulated genes were mainly enriched in cellular response to zinc ion and humoral immune response. In the CC group, up-regulated genes were mainly enriched in collagen-containing ECM and ECM components while down-regulated genes mainly enriched in blood microparticle. In the MF group, up-regulated genes were mainly enriched in ECM structural constituents while down-regulated genes were mainly enriched in pattern binding. Taken together, these data suggest that those identified DEGs are mainly enriched in ECM-related items affecting the BP of negative regulation of growth, humoral immune response and so on.

**TABLE 2:** Significantly enriched GO terms of DEGs associated with HCC with adjust *p*-value < 0.01.

**Expression**	**Category**	**Term**	**Count**	***p*-value**	**Adj *p*-value**
Up-regulated	CC	GO:0062023∼collagen-containing extracellular matrix	9	<0.001	<0.001
	CC	GO:0044420∼extracellular matrix component	5	<0.001	<0.001
	CC	GO:0098644∼complex of collagen trimers	4	<0.001	<0.001
	CC	GO:0031012∼extracellular matrix	9	<0.001	<0.001
	CC	GO:0005581∼collagen trimer	5	<0.001	<0.001
	CC	GO:0005788∼endoplasmic reticulum lumen	7	<0.001	<0.001
	CC	GO:0005604∼basement membrane	4	<0.001	<0.001
	CC	GO:0042470∼melanosome	4	<0.001	<0.001
	CC	GO:0048770∼pigment granule	4	<0.001	<0.001
	CC	GO:0005583∼fibrillar collagen trimer	2	<0.001	0.001
	CC	GO:0098643∼banded collagen fibril	2	<0.001	0.001
	MF	GO:0030020∼extracellular matrix structural constituent conferring tensile strength	5	<0.001	<0.001
	MF	GO:0005201∼extracellular matrix structural constituent	5	<0.001	0.001
	MF	GO:0048407∼platelet-derived growth factor binding	2	<0.001	0.008
Down-regulated	BP	GO:0071294∼cellular response to zinc ion	5	<0.001	<0.001
	BP	GO:0010043∼response to zinc ion	6	<0.001	<0.001
	BP	GO:0006956∼complement activation	7	<0.001	<0.001
	BP	GO:0072376∼protein activation cascade	7	<0.001	<0.001
	BP	GO:0071276∼cellular response to cadmium ion	4	<0.001	0.001
	BP	GO:0001867∼complement activation, lectin pathway	3	<0.001	0.001
	BP	GO:0006959∼humoral immune response	7	<0.001	0.003
	BP	GO:0046686∼response to cadmium ion	4	<0.001	0.004
	BP	GO:0010460∼positive regulation of heart rate	3	<0.001	0.008
	BP	GO:0010038∼response to metal ion	7	<0.001	0.008
	CC	GO:0072562∼blood microparticle	5	<0.001	0.008
	MF	GO:0001871∼pattern binding	3	<0.001	0.004
	MF	GO:0030247∼polysaccharide binding	3	<0.001	0.004
	MF	GO:1901681∼sulfur compound binding	6	<0.001	0.005
	MF	GO:0050662∼coenzyme binding	6	<0.001	0.008

*BP, biological process; CC, cellular component; MF, molecular function.*

### Signaling Pathway Enrichment Analysis

To understand the biological changes during HCC pathogenesis, we performed pathway enrichment analysis using KEGG and Reactome. KEGG pathways enrichment analysis showed that those candidate DEGs were primarily enriched in protein digestion and absorption, mineral absorption, and ECM-receptor interaction (Figure 2B). Among them, up-regulated genes were mainly enriched in protein digestion and absorption and ECM-receptor interaction while down-regulated genes were mainly enriched in mineral absorption and metabolic pathways (Table 3). Furthermore, Reactome pathway enrichment analysis showed that the DEGs were enriched in collagen chain trimerization, collagen degradation, metallothioneins bind metals, and response to metal ions (Supplementary Table S4). Among them, up-regulated genes were primarily enriched in collagen chain trimerization, assembly of collagen fibrils and other multimeric structures and collagen degradation, while down-regulated genes were enriched in metallothioneins bind metals, response to metal ions and ficolins bind to repetitive carbohydrate structures on the target cell surface (Table 4).

**TABLE 3:** KEGG pathway enrichment analysis of DEGs in HCC.

**Expression**	**KEGG Term**	**Count**	***p*-value**	**Adj *p*-value**
Up-regulated	hsa04974∼Protein digestion and absorption	6	<0.001	<0.001
	hsa04512∼ECM-receptor interaction	4	<0.001	0.003
	hsa04964∼Proximal tubule bicarbonate reclamation	2	0.002	0.028
	hsa04510∼Focal adhesion	4	0.002	0.028
	hsa04151∼*PI3K/Akt* signaling pathway	5	0.002	0.028
	hsa04933∼*AGE-RAGE* signaling pathway in diabetic complications	3	0.003	0.028
	hsa05146∼Amoebiasis	3	0.003	0.028
	hsa04926∼Relaxin signaling pathway	3	0.005	0.048
Down-regulated	hsa04978∼Mineral absorption	5	<0.001	0.001
	hsa00640∼Propanoate metabolism	3	0.001	0.025

**TABLE 4:** Pathways enriched in Reactome analysis of DEGs in HCC (Adj *p*-value < 0.01).

**Expression**	**Pathway name**	**Count**	***p*-value**	**Adj *p*-value**
Up-regulated	R-HSA-8948216∼Collagen chain trimerization	5	<0.001	<0.001
	R-HSA-2022090∼Assembly of collagen fibrils and other multimeric structures	5	<0.001	<0.001
	R-HSA-1442490∼Collagen degradation	5	<0.001	<0.001
	R-HSA-1650814∼Collagen biosynthesis and modifying enzymes	5	<0.001	<0.001
	R-HSA-216083∼Integrin cell surface interactions	6	<0.001	<0.001
	R-HSA-1474228∼Degradation of the extracellular matrix	6	<0.001	<0.001
	R-HSA-1474290∼Collagen formation	5	<0.001	<0.001
	R-HSA-3000171∼Non-integrin membrane-ECM interactions	4	<0.001	<0.001
	R-HSA-1474244∼Extracellular matrix organization	6	<0.001	<0.001
	R-HSA-2214320∼Anchoring fibril formation	3	<0.001	0.001
	R-HSA-422475∼Axon guidance	8	<0.001	0.002
	R-HSA-8985801∼Regulation of cortical dendrite branching	1	<0.001	0.002
	R-HSA-2243919∼Crosslinking of collagen fibrils	3	<0.001	0.002
	R-HSA-186797∼Signaling by *PDGF*	4	<0.001	0.003
	R-HSA-8941333∼*RUNX2* regulates genes involved in differentiation of myeloid cells	1	<0.001	0.004
	R-HSA-3000178∼ECM proteoglycans	4	<0.001	0.004
	R-HSA-69205∼G1/S-Specific Transcription	2	0.001	0.009
	R-HSA-3769402∼Deactivation of the beta-catenin transactivating complex	3	0.001	0.009
	R-HSA-419037∼*NCAM1* interactions	3	0.001	0.009
	R-HSA-8949275∼*RUNX3* Regulates Immune Response and Cell Migration	1	0.001	0.009
	R-HSA-8939246∼*RUNX1* regulates transcription of genes involved in differentiation of myeloid cells	1	0.001	0.010
	R-HSA-3000480∼Scavenging by Class A Receptors	3	0.001	0.010
Down-regulated	R-HSA-5661231∼Metallothioneins bind metals	5	<0.001	<0.001
	R-HSA-5660526∼Response to metal ions	5	<0.001	<0.001
	R-HSA-2855086∼Ficolins bind to repetitive carbohydrate structures on the target cell surface	3	<0.001	<0.001
	R-HSA-166662∼Lectin pathway of complement activation	3	<0.001	<0.001
	R-HSA-166658∼Complement cascade	6	<0.001	0.003

### Key Candidate Genes and Pathways Identified by DEGs PPI and Modular Analysis

**FIGURE 3:**
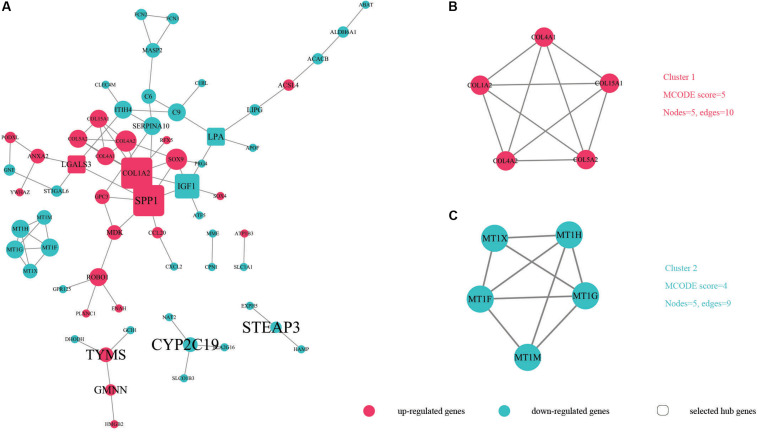
The protein–protein interaction (PPI) of DEGs exported from STRING is visualized using Cytoscape software. Up-regulated DEGs are shown in red while down-regulated DEGs are shown in green dots. **(A)** 63 nodes and 78 edges are displayed. Larger node sizes correspond to higher degrees of DEGs. Label font sizes are shown in black from small to large according to the Betweenness centrality (from low to high). The hub genes we selected are emphasized and shown in the shape of round rectangles. **(B,C)** Module 1, module 2 and their related specifications determined by MCODE plug-in app in Cytoscape software. Circles represent genes and the lines between genes indicate the gene-encoded PPIs.

In order to identify key candidate genes, 63 DEGs (24 up-regulated genes and 39 down-regulated genes) were filtered into the PPI network complex, including 63 nodes and 78 edges. Among the 63 nodes, only genes ranking in top 10 of both degrees (the number of interactions of each node) and betweenness centrality (degree of impact on interactions between other nodes in the network) parameters were recognized as hub genes. Finally, five genes *SPP1*, *COL1A2*, *IGF1*, *LGALS3*, and *LPA* were selected (Figure 3A and Table 5). Utilizing MCODE plug-in app in cytoscape, two modules were applied for further KEGG pathway enrichment analysis. Module 1 consisted of 5 nodes and 10 edges with genes enriched in protein digestion and absorption, ECM-receptor interaction, amoebiasis and focal adhesion. Module 2 consisted of five nodes and nine edges with the genes mainly enriched in mineral absorption (Figures 3B,C and Supplementary Table S5).

**TABLE 5:** Top 10 most degree values and betweenness centrality hub genes between HCC and normal samples.

**Genes**	**Expression**	**Betweenness centrality**	**Genes**	**Expression**	**Degree**
*CYP2C19*	Down	1	***SPP1***	Up	8
*STEAP3*	Down	1	***COL1A2***	Up	8
*TYMS*	Up	0.83333333	***IGF1***	Down	6
***SPP1***	Up	0.50873984	*SOX9*	Up	5
*GMNN*	Up	0.5	*COL4A2*	Up	5
***LPA***	Down	0.30264228	***LPA***	Down	4
***IGF1***	Down	0.29329268	***LGALS3***	Up	4
***LGALS3***	Up	0.24573171	*C9*	Down	4
***COL1A2***	Up	0.19756098	*SERPINA10*	Down	4
*MDK*	Up	0.1804878	*ROBO1*	Up	4

*Genes were ranked by betweenness centrality and degree, respectively. The genes in red are the shared genes with the two analysis methods.*

### Hub Genes and Associations With Clinical Outcome

**FIGURE 4:**
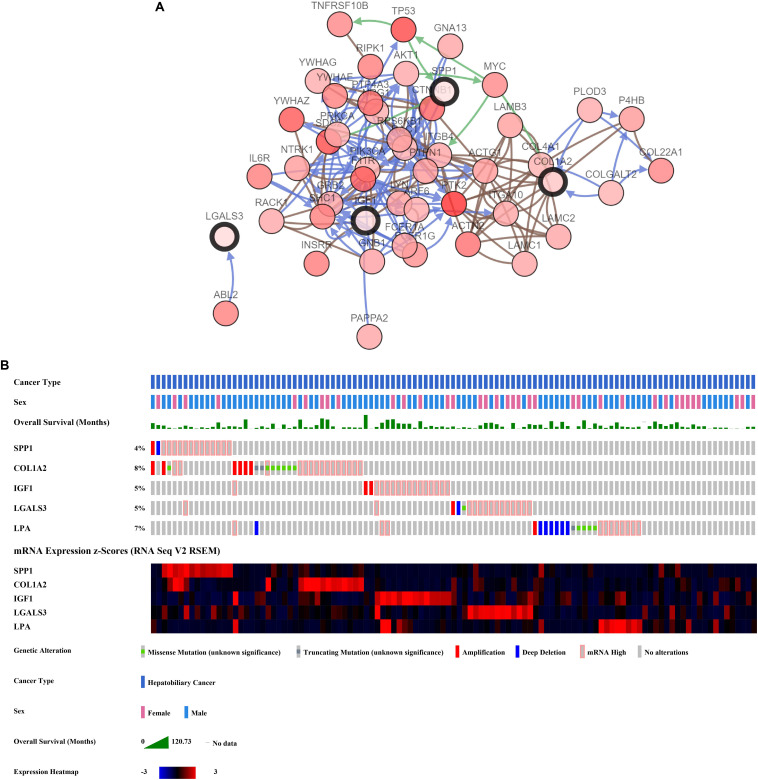
Analysis of hub genes. **(A)** Hub genes and their co-expressed genes are analyzed using cBioPortal. Hub genes are represented with a thick border. Darker red indicates increased frequency of alteration in HCC. The blue connection indicates that the first protein controlled a reaction that changes the state of the second protein; the red connection suggests that the proteins belongs to members of the same complex. The green arrows represent “Controls expression of.” The gray arrows represent “In complex with.” **(B)** Genetic alteration analysis toward these five genes, overall survivals are also shown.

**FIGURE 5:**
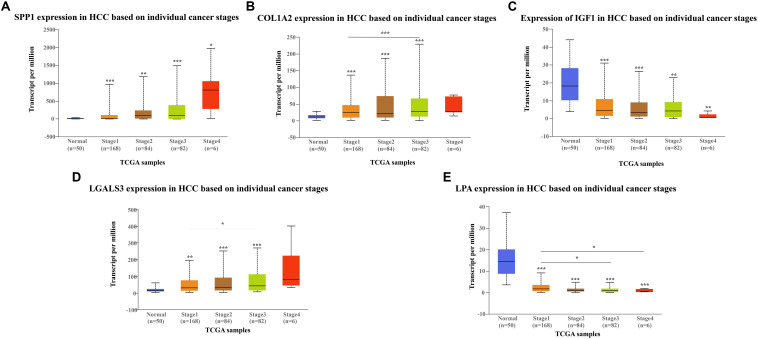
Expression of hub genes in different HCC stages in TGCA database: **(A)** *SPP1*, **(B)** *COL1A2*, **(C)** *IGF1*, **(D)** *LGALS3*, and **(E)** *LPA*. *SPP1*, *COL1A2*, and *LGAlS3* are overexpressed in HCC tissues while *IGF1* and *LPA* are downregulated in HCC tissues compared to the control.

**FIGURE 6:**
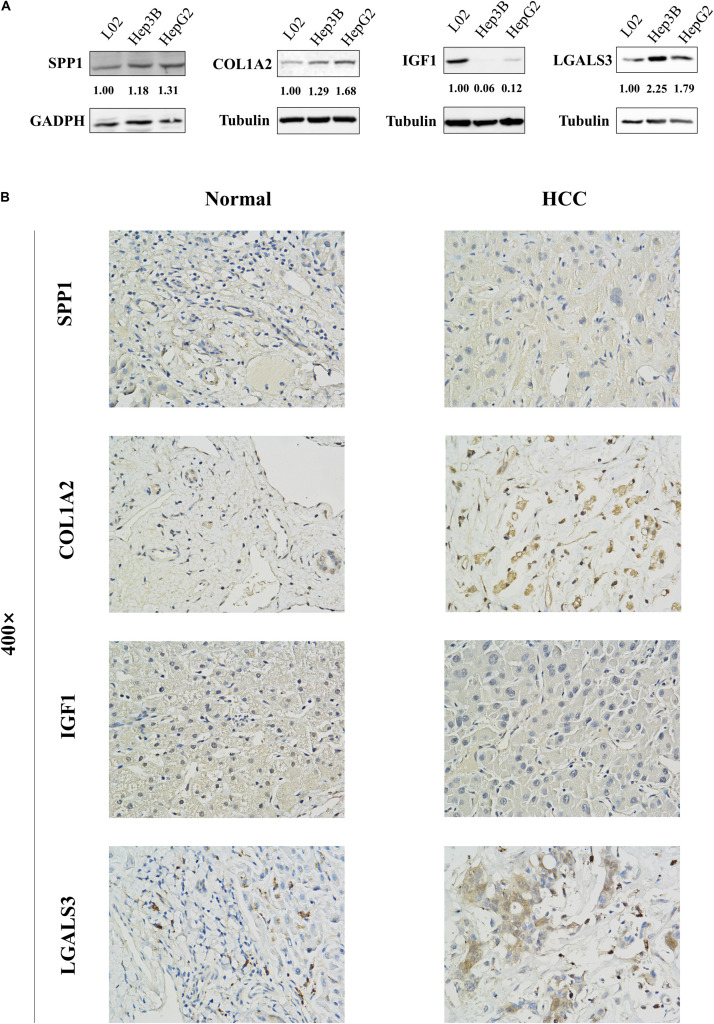
Protein expression levels of these hub genes in normal liver cell line L02 and HCC cell lines Hep3B and HepG2 were examined by western blot analysis. The bands were analyzed and normalized using the GADPH or β-Tubulin values and processed by Image J software. **(A)** The representative cases of HCC patient tissues were detected by **(B)** Immunohistochemistry. The western blot and Immunohistochemistry results showed that SPP1, COL1A2, and LGALS3 are overexpressed in HCC samples while IGF1 is downregulated in HCC samples.

**FIGURE 7:**
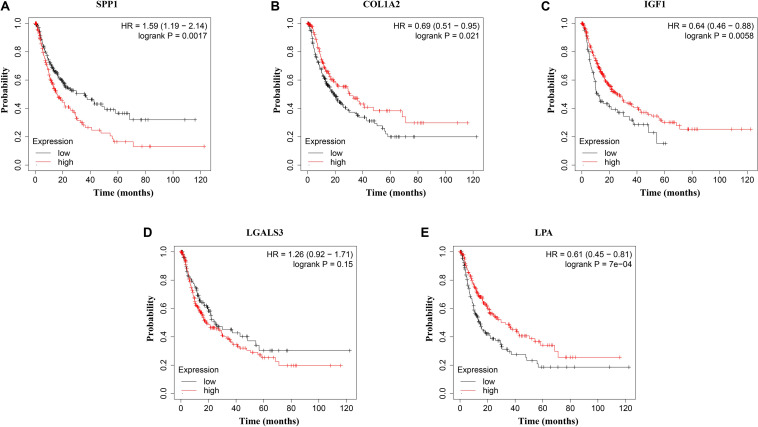
Disease-free survival (DFS) analysis of **(A)** *SPP1*, **(B)** *COL1A2*, **(C)** *IGF1*, **(D)** *LGALS3*, and **(E)** *LPA* in HCC patients. HCC patients with high expressions of *COL1A2*, *IGF1*, and *LPA* as well as low expression of *SPP1* were found to be associated with the improved DFS (*p* = 0.0017, *p* = 0.0021, *p* = 0.0058, and *p* = 7e^–04^, respectively).

**FIGURE 8:**
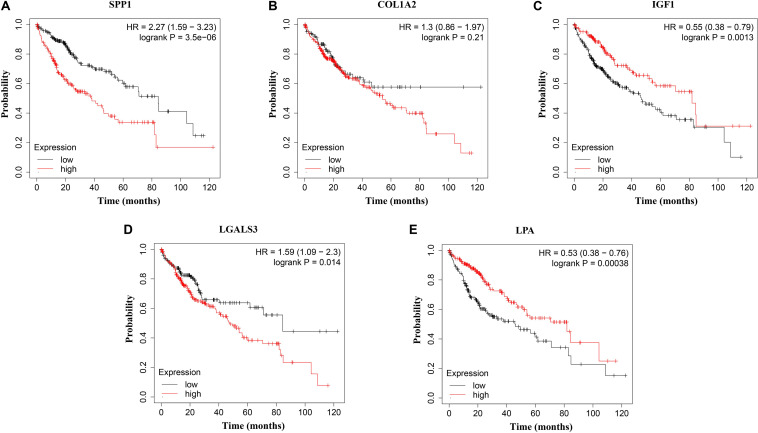
Overall survival (OS) analysis of **(A)** *SPP1*, **(B)** *COL1A2*, **(C)** *IGF1*, **(D)** *LGALS3*, and **(E)** *LPA* in HCC patients. High expression of *SPP1* and *LGALS3* were linked with the disfavored OS (*p* = 3.5e^–06^ and *p* = 0.014, respectively) **(A,D)**, while high expression of *IGF1* and *LPA* were associated with improved OS (*p* = 0.0013 and *p* = 0.00038, respectively).

The network of hub genes constructed by cBioPortal contained 55 nodes, including five query genes (five hub genes) and the 50 most frequently altered neighbor genes (Figure 4A). After visualizing BP using BiNGO in Cytoscape software (Supplementary Figure S1), genetic alteration analysis of five hub genes in TCGA HCC patients was performed in the cBioPortal database. The hub genes *SPP1*, *IGF1*, *LGALS3*, *LPA*, and *COL1A2* were altered in 4, 5, 5, 7, and 8% in a total population of HCC patients respectively, without significantly discrepancy in both sexes (Figure 4B). TCGA data analysis showed that *SPP1*, *COL1A2*, and LGALS3 are more highly expressed in HCC regardless of stages compared with normal tissues, while *IGF1* and *LPA* were low expressed (Figure 5). Further western blot analysis showed that the protein expression levels of *SPP1*, *COL1A2*, and *LGALS3* were highly expressed in HCC cell lines while IGF1 was down-regulated in HCC cell lines (Figure 6A). IHC analysis of HCC patient tissues showed similar results as western blot analysis (Figure 6B). The online human protein atlas showed the LPA protein expression was higher in normal liver tissues than in HCC tissues. For the identified five top hub genes, HCC patients with high expressions of *COL1A2*, *IGF1*, and *LPA* as well as low expression of *SPP1* were found to be associated with the improved DFS (*p* = 0.0017, *p* = 0.0021, *p* = 0.0058, and *p* = 7e^–04^, respectively) (Figure 7). High expression of *SPP1* and *LGALS3* were linked with the disfavored OS (*p* = 3.5e^–06^ and *p* = 0.014, respectively) (Figures 8A,D), while high expression of *IGF1* and *LPA* were associated with improved OS (*p* = 0.0013 and *p* = 0.00038, respectively) (Figures 8C,E). However, expression of *COL1A2* didn’t show a significant correlation with clinical outcome (*p* = 0.21) (Figure 8B).

## Discussion

Hepatocellular carcinoma remains an aggressive form of cancer worldwide with high incidence and morbidity. Therefore, substantial efforts have been made to unveil mutational processes, pathogenesis and possible mechanisms underlying treatment resistance in order to expand the therapeutic landscape of this disease (Llovet et al., 2018; Cheng et al., 2019). However, most of these studies were based on single institutions with limited sample size, restricting the power to identify potential meaningful therapeutic targets (Zhang C. et al., 2017; Li et al., 2019; Zhang et al., 2019). Different HCC studies showed different results for different datasets chosen. In previous studies, some only chose one dataset and others chose datasets without performing explicit infiltration, leading to totally different outcome (Zhang C. et al., 2017; Li et al., 2019; Zhang et al., 2019). Here, we conducted an integrative analysis from four microarray datasets of HCC screened in Oncomine database and downloaded in GEO database to describe key candidate genes and pathways associated with clinical outcome in HCC patients.

In the present study, a total of 89 DEGs were identified between HCC and normal tissues, including 31 up-regulated genes and 58 down-regulated genes. Up-regulated DEGs of HCC were found to be enriched in GO categories such as epithelial tube morphogenesis, ECM organization, and positive regulation of leukocyte migration, and dysregulation of these processes have been found to contribute to several pathological conditions including cancer and may lead to disfavored clinical outcomes (Payne and Huang, 2013; Bonnans et al., 2014). While down-regulated genes were associated with GO categories such as cellular response to zinc ion where members of metallothionein family (*MT1M*, *MT1H*, *MT1X*, *MT1G*, and *MT1F*) play important roles in carcinogenesis of various cancer types (Si and Lang, 2018). KEGG pathway enrichment analysis demonstrated that up-regulated genes were significantly enriched in protein digestion and absorption, ECM-receptor interaction and PI3K/Akt signaling pathway while down-regulated genes were enriched in mineral absorption and metabolic pathways, and all those are significant pathways in various cancer types been reported previously (Boroughs and DeBerardinis, 2015; Dimitrova and Arcaro, 2015; Wang S.S. et al., 2017; Slattery et al., 2018). Intriguingly, a host of altered genes were found to be associated with ECM related pathways. The ECM, an extensive part of the microenvironment in all tissues, providing a physical scaffold for its surrounding cells, bind growth factors and regulate cell behavior, plays a vital part in tumor progression (Kalluri, 2016; Nissen et al., 2019).

We also constructed PPI network and identified five hub genes *SPP1*, *COL1A2*, *IGF1*, *LPA*, and *LGALS3* as key candidate genes potentially linked with pathogenesis of HCC. Their co-expressed genes were then analyzed using cBioPortal online platform. The results contained five query genes and the 50 most frequently altered neighbor genes. Among the five hub genes, *SPP1*, *COL1A2*, *IGF1*, and *LGALS3* and their co-expressed genes constructed a network. *LPA* and its co-expressed genes were isolated from the main network and didn’t have directly interaction with it. That’s why only four out of five hub genes contained in Figure 4A. Both SPP1 and COL1A2 are members belonging to PI3K/Akt signaling pathway and ECM-receptor interaction pathway regulating cell growth (Fang et al., 2017) and drug resistance (Zhang et al., 2016). SPP1, also known as osteopontin, has been reported to have the capability of regulating cell behaviors (Rowe et al., 2014). Previous data also showed that targeting SPP1 could inhibit gastric cancer cell epithelial–mesenchymal transition through inhibition of the PI3K/AKT signaling pathway (Song et al., 2019). In lung adenocarcinoma, SPP1 was found to up-regulate PD-L1 and subsequently facilitated the escape of immunity (Zhang Y. et al., 2017). These studies demonstrated that SPP1 was highly associated with the cancer invasion and progression, suggesting its potential to serve as a biomarker and target for the diagnosis and treatment of HCC. COL1A2, a member of group I collagen family, has once been reported as a target of Let-7g thus inhibiting cell migration in HCC (Ji et al., 2010) and gastric cancer cell proliferation (Ao et al., 2018). In 2018, a study found that the silencing of *COL1A2* could inhibit the proliferation, migration, and invasion of gastric cancer through regulating PI3K/AKT signaling pathway, revealing the potency of COL1A2 in HCC (Ao et al., 2018). IGF1, insulin-like growth factor 1, has the capability of maintaining the stemness in HCC, and its role of serving as an anticancer target has been confirmed by several studies (Kaseb et al., 2011, 2014; Chen and Sharon, 2013; Bu et al., 2014). IGF1 and IGF2 comprise of the IGF family, contributing largely to the activation of the PI3K/Akt signaling pathway, which was also found dysregulated by the KEGG analysis, thus enhancing the cancerogenesis of HCC (Kasprzak et al., 2017). LPA is lipoprotein A, a special kind of low-density lipoprotein, has shown the evidence of causing inflammation and regulating HCC cell proliferation (Pirro et al., 2017; Xu et al., 2017). Patients with HCC showed a statistically significant serum LPA level higher than the healthy subjects, indicating its important role in HCC patients (Malaguarnera et al., 2017). *LGALS3*, which encodes the Galectin-3 protein, is regarded as a guardian of the tumor microenvironment (Ruvolo, 2016). Recent studies have shown that *LGALS3* is tightly associated with several malignancies such as Hodgkin’s lymphoma (Koh et al., 2014), acute myeloid leukemia (Cheng et al., 2013), and HCC (Song et al., 2014). More importantly, *LGALS3* could increase the metastatic potential of breast cancer, might accounting for the metastatic potential of HCC (Pereira et al., 2019). Through validation in western blot and IHC assays, we found that the protein expression of these five hub genes was in accordance with their mRNA expression in HCC patient tissues. Strikingly, LPA has not been tested by western blot and IHC assays due to its large molecular weight of 501 kDa, exerting huge difficulty in performing these assays. Previous studies have shown that these genes are implicated in the tumorigenesis and transformation (Oates et al., 1997; Orsó and Schmitz, 2017; Wang Y.A. et al., 2017; Diao et al., 2018; Ma et al., 2019). In our study, correlations of *SPP1*, *COL1A2*, *IGF1*, *LPA*, and *LGALS3* with patient prognosis highlight the importance of these five genes as potential biomarkers to stratify HCC patients as well as potential therapeutic targets, but concrete roles of these genes need further investigation. In the future studies, we will develop knockdown and overexpression HCC cell lines and mouse models of these five hub genes to demonstrate their importance in the progression of HCC *in vitro* and *in vivo*.

Taken together, this study integrated four datasets to screen for reliable and accurate biomarkers of HCC and demonstrated that several pathways are altered. Several hub genes with the expression levels have significantly associated with clinical outcome in HCC patients. Further functional study on the mechanisms of those genes leading to HCC is under way.

## Data Availability Statement

Publicly available datasets were analyzed in this study. This data can be found here: https://www.oncomine.org.

## Ethics Statement

The studies involving human participants were reviewed and approved by the Cancer Hospital of the University of Chinese Academy of Sciences; Zhejiang Cancer Hospital. The patients/participants provided their written informed consent to participate in this study.

## Author Contributions

ST, WZ, and J-JQ: conceptualization and supervision. YL, JY, SM, and X-DC: investigations. YL, JY, KQ, CK, and RC: methodology. YL and JY: data curation. YL and RC: original draft writing. YL, RC, and J-JQ: writing review and editing. All authors contributed to the article and approved the submitted version.

## Conflict of Interest

KQ was employed by the company Accenture Applied Intelligence, ASEAN. The remaining authors declare that the research was conducted in the absence of any commercial or financial relationships that could be construed as a potential conflict of interest.

## Footnotes

1 https://www.oncomine.org/

2 https://www.ncbi.nlm.nih.gov/geo/geo2r/

3 https://reactome.org/

4 https://string-db.org/

5 https://david.ncifcrf.gov/

6 https://www.cbioportal.org/

7 http://ualcan.path.uab.edu/analysis.html

8 http://www.kmplot.com/

9 https://www.proteinatlas.org/

10 https://www.r-project.org/

## Supplementary Material

FIGURE S1 The biological process analysis of hub genes using BINGO.

TABLE S1 The DEGs identified in each dataset.

TABLE S2 Information of DEGs screened from each dataset.

TABLE S3 31 consistently up-regulated and 58 down-regulated DEGs.

TABLE S4 Reactome pathway enrichment analysis.

TABLE S5 KEGG pathway enrichment analysis of modules.

## References

[B1] Ally A. Balasundaram M. Carlsen R. Chuah E. Clarke A. Dhalla N. (2017). Comprehensive and integrative genomic characterization of hepatocellular carcinoma. *Cell* 169 1327–1341.e1323. 28622513 10.1016/j.cell.2017.05.046 PMC5680778

[B2] Ao R. Guan L. Wang Y. Wang J.-N. (2018). Silencing of COL1A2, COL6A3, and THBS2 inhibits gastric cancer cell proliferation, migration, and invasion while promoting apoptosis through the PI3k-Akt signaling pathway. *J. Cell. Biochem.* 119 4420–4434. 10.1002/jcb.26524 29143985

[B3] Aravalli R. N. Cressman E. N. Steer C. J. (2013). Cellular and molecular mechanisms of hepatocellular carcinoma: an update. *Arch. Toxicol.* 87 227–247. 10.1007/s00204-012-0931-2 23007558

[B4] Bader G. D. Hogue C. W. (2003). An automated method for finding molecular complexes in large protein interaction networks. *BMC Bioinformatics* 4:2. 10.1186/1471-2105-4-2 12525261 PMC149346

[B5] Bonnans C. Chou J. Werb Z. (2014). Remodelling the extracellular matrix in development and disease. *Nat. Rev. Mol. Cell Biol.* 15 786–801. 10.1038/nrm3904 25415508 PMC4316204

[B6] Boroughs L. K. DeBerardinis R. J. (2015). Metabolic pathways promoting cancer cell survival and growth. *Nat. Cell Biol.* 17 351–359. 10.1038/ncb3124 25774832 PMC4939711

[B7] Bray F. Ferlay J. Soerjomataram I. Siegel R. L. Torre L. A. Jemal A. (2018). Global cancer statistics 2018: GLOBOCAN estimates of incidence and mortality worldwide for 36 cancers in 185 countries. *CA Cancer J. Clin.* 68 394–424. 10.3322/caac.21492 30207593

[B8] Bu Y. Jia Q. A. Ren Z. G. Zhang J. B. Jiang X. M. Liang L. (2014). Maintenance of stemness in oxaliplatin-resistant hepatocellular carcinoma is associated with increased autocrine of IGF1. *PLoS One* 9:e89686. 10.1371/journal.pone.0089686 24632571 PMC3954560

[B9] Chandrashekar D. S. Bashel B. Balasubramanya S. A. H. Creighton C. J. Ponce-Rodriguez I. Chakravarthi B. (2017). UALCAN: a portal for facilitating tumor subgroup gene expression and survival analyses. *Neoplasia* 19 649–658. 10.1016/j.neo.2017.05.002 28732212 PMC5516091

[B10] Chen H. X. Sharon E. (2013). IGF-1R as an anti-cancer target–trials and tribulations. *Chin. J. Cancer* 32 242–252. 10.5732/cjc.012.10263 23601239 PMC3845553

[B11] Cheng C. L. Hou H. A. Lee M. C. Liu C. Y. Jhuang J. Y. Lai Y. J. (2013). Higher bone marrow LGALS3 expression is an independent unfavorable prognostic factor for overall survival in patients with acute myeloid leukemia. *Blood* 121 3172–3180. 10.1182/blood-2012-07-443762 23449638

[B12] Cheng H. Sun G. Chen H. Li Y. Han Z. Li Y. (2019). Trends in the treatment of advanced hepatocellular carcinoma: immune checkpoint blockade immunotherapy and related combination therapies. *Am. J. Cancer Res.* 9 1536–1545. 31497341 PMC6726979

[B13] Crawley J. J. Furge K. A. (2002). Identification of frequent cytogenetic aberrations in hepatocellular carcinoma using gene-expression microarray data. *Genome Biol.* 3:research0075.0071. 10.1186/gb-2002-3-12-research0075 PMC151177 12537564

[B14] Diao B. Liu Y. Xu G.-Z. Zhang Y. Xie J. Gong J. (2018). The role of galectin-3 in the tumorigenesis and progression of pituitary tumors. *Oncol. Lett.* 15 4919–4925. 10.3892/ol.2018.7931 29545898 PMC5840766

[B15] Dimitrova V. Arcaro A. (2015). Targeting the PI3K/AKT/mTOR signaling pathway in medulloblastoma. *Curr. Mol. Med.* 15 82–93. 10.2174/1566524015666150114115427 25601471

[B16] Duan Y. (2019a). *Existence Computation: Relationship Defined Everything Underlying Semantic Computation.* Toyama, 139–144.

[B17] Duan Y. (2019b). “Towards a periodic table of conceptualization and formalization on concepts of state, style, structure, pattern, framework, architecture, service, etc., based on existence computation and relationship defined everything of semantic,” in *Proceedings of the 20th IEEE/ACIS International Conference on Software Engineering, Artificial Intelligence, Networking and Parallel/Distributed Computing (SNPD)*, Toyama.

[B18] Duan Y. Lu Z. Zhou Z. Sun X. Wu J. (2019a). Data privacy protection for edge computing of smart city in a DIKW architecture. *Eng. Appl. Artif. Intell.* 81 323–335. 10.1016/j.engappai.2019.03.002

[B19] Duan Y. Sun X. Che H. Cao C. Li Z. Yang X. (2019b). Modeling data, information and knowledge for security protection of hybrid IoT and edge resources. *IEEE Access* 7 99161–99176. 10.1109/access.2019.2931365

[B20] Fabregat A. Jupe S. Matthews L. Sidiropoulos K. Gillespie M. Garapati P. (2018). The reactome pathway knowledgebase. *Nucleic Acids Res.* 46 D649–D655. 10.1093/nar/gkx1132 29145629 PMC5753187

[B21] Fang X. Yang D. Luo H. Wu S. Dong W. Xiao J. (2017). SNORD126 promotes HCC and CRC cell growth by activating the PI3K-AKT pathway through FGFR2. *J. Mol. Cell Biol.* 9 243–255. 10.1093/jmcb/mjw048 27913571

[B22] Gao J. Aksoy B. A. Dogrusoz U. Dresdner G. Gross B. Sumer S. O. (2013). Integrative analysis of complex cancer genomics and clinical profiles using the cBioPortal. *Sci. Signal.* 6:l1. 10.1126/scisignal.2004088 23550210 PMC4160307

[B23] Hoshida Y. Nijman S. M. Kobayashi M. Chan J. A. Brunet J.-P. Chiang D. Y. (2009). Integrative transcriptome analysis reveals common molecular subclasses of human hepatocellular carcinoma. *Cancer Res.* 69 7385–7392. 10.1158/0008-5472.can-09-1089 19723656 PMC3549578

[B24] Huang da W. Sherman B. T. Lempicki R. A. (2009). Systematic and integrative analysis of large gene lists using DAVID bioinformatics resources. *Nat. Protoc.* 4 44–57. 10.1038/nprot.2008.211 19131956

[B25] Ji J. Zhao L. Budhu A. Forgues M. Jia H. L. Qin L. X. (2010). Let-7g targets collagen type I alpha2 and inhibits cell migration in hepatocellular carcinoma. *J. Hepatol.* 52 690–697. 10.1016/j.jhep.2009.12.025 20338660 PMC2862772

[B26] Jin B. Wang W. Du G. Huang G. Han L. Tang Z. (2015). Identifying hub genes and dysregulated pathways in hepatocellular carcinoma. *Eur. Rev. Med. Pharmacol. Sci.* 19 592–601. 25753876

[B27] Kalluri R. (2016). The biology and function of fibroblasts in cancer. *Nat. Rev. Cancer* 16 582–598. 10.1038/nrc.2016.73 27550820

[B28] Kanehisa M. Goto S. (2000). KEGG: kyoto encyclopedia of genes and genomes. *Nucleic Acids Res.* 28 27–30. 10.1093/nar/28.1.27 10592173 PMC102409

[B29] Kanehisa M. Goto S. Sato Y. Furumichi M. Tanabe M. (2012). KEGG for integration and interpretation of large-scale molecular data sets. *Nucleic Acids Res.* 40 D109–D114. 10.1093/nar/gkr988 22080510 PMC3245020

[B30] Kanehisa M. Sato Y. Kawashima M. Furumichi M. Tanabe M. (2016). KEGG as a reference resource for gene and protein annotation. *Nucleic Acids Res.* 44 D457–D462. 10.1093/nar/gkv1070 26476454 PMC4702792

[B31] Kaseb A. O. Morris J. S. Hassan M. M. Siddiqui A. M. Lin E. Xiao L. (2011). Clinical and prognostic implications of plasma insulin-like growth factor-1 and vascular endothelial growth factor in patients with hepatocellular carcinoma. *J. Clin. Oncol.* 29 3892–3899. 10.1200/jco.2011.36.0636 21911725 PMC3189091

[B32] Kaseb A. O. Xiao L. Hassan M. M. Chae Y. K. Lee J. S. Vauthey J. N. (2014). Development and validation of insulin-like growth factor-1 score to assess hepatic reserve in hepatocellular carcinoma. *J. Nat. Cancer Inst.* 106:dju088. 10.1093/jnci/dju088 24815863 PMC4085880

[B33] Kasprzak A. Kwasniewski W. Adamek A. Gozdzicka-Jozefiak A. (2017). Insulin-like growth factor (IGF) axis in cancerogenesis. *Mutat. Res. Rev. Mutat. Res.* 772 78–104. 10.1016/j.mrrev.2016.08.007 28528692

[B34] Koh Y. W. Jung S. J. Park C. S. Yoon D. H. Suh C. Huh J. (2014). LGALS3 as a prognostic factor for classical Hodgkin’s lymphoma. *Mod. Pathol.* 27 1338–1344. 10.1038/modpathol.2014.38 24603587

[B35] Li C. Zhou D. Jiang X. Liu M. Tang H. Mei Z. (2019). Identifying hepatocellular carcinoma-related hub genes by bioinformatics analysis and CYP2C8 is a potential prognostic biomarker. *Gene* 698 9–18. 10.1016/j.gene.2019.02.062 30825595

[B36] Llovet J. M. Montal R. Sia D. Finn R. S. (2018). Molecular therapies and precision medicine for hepatocellular carcinoma. *Nat. Rev. Clin. Oncol.* 15 599–616. 10.1038/s41571-018-0073-4 30061739 PMC12452113

[B37] Ma H.-P. Chang H.-L. Bamodu O. A. Yadav V. K. Huang T.-Y. Wu A. T. H. (2019). Collagen 1A1 (COL1A1) is a reliable biomarker and putative therapeutic target for hepatocellular carcinogenesis and metastasis. *Cancers* 11:786. 10.3390/cancers11060786 31181620 PMC6627889

[B38] Maere S. Heymans K. Kuiper M. (2005). BiNGO: a cytoscape plugin to assess overrepresentation of gene ontology categories in biological networks. *Bioinformatics* 21 3448–3449. 10.1093/bioinformatics/bti551 15972284

[B39] Malaguarnera G. Catania V. E. Francaviglia A. Malaguarnera M. Drago F. Motta M. (2017). Lipoprotein(a) in patients with hepatocellular carcinoma and portal vein thrombosis. *Aging Clin. Exp. Res.* 29(Suppl. 1) 185–190. 10.1007/s40520-016-0653-z 27822883

[B40] Marengo A. Rosso C. Bugianesi E. (2016). Liver cancer: connections with obesity, fatty liver, and cirrhosis. *Annu. Rev. Med.* 67 103–117. 10.1146/annurev-med-090514-013832 26473416

[B41] Mas V. R. Maluf D. G. Archer K. J. Yanek K. Kong X. Kulik L. (2009). Genes involved in viral carcinogenesis and tumor initiation in hepatitis C virus-induced hepatocellular carcinoma. *Mol. Med.* 15 85–94. 10.2119/molmed.2008.00110 19098997 PMC2605622

[B42] Nissen N. I. Karsdal M. Willumsen N. (2019). Collagens and Cancer associated fibroblasts in the reactive stroma and its relation to Cancer biology. *J. Exp. Clin. Cancer Res.* 38:115. 10.1186/s13046-019-1110-6 30841909 PMC6404286

[B43] Oates A. J. Barraclough R. Rudland P. S. (1997). The role of osteopontin in tumorigenesis and metastasis. *Invasion Metastasis* 17 1–15. 9425320

[B44] Orsó E. Schmitz G. (2017). Lipoprotein(a) and its role in inflammation, atherosclerosis and malignancies. *Clin. Res. Cardiol. Suppl.* 12(Suppl. 1) 31–37. 10.1007/s11789-017-0084-1 28188431 PMC5352764

[B45] Oweira H. Petrausch U. Helbling D. Schmidt J. Mehrabi A. Schob O. (2017). Prognostic value of site-specific extra-hepatic disease in hepatocellular carcinoma: a SEER database analysis. *Exp. Rev. Gastroenterol. Hepatol.* 11 695–701. 10.1080/17474124.2017.1294485 28276812

[B46] Payne L. S. Huang P. H. (2013). The pathobiology of collagens in glioma. *Mol. Cancer Res.* 11 1129–1140. 10.1158/1541-7786.MCR-13-0236 23861322 PMC3836242

[B47] Pereira J. X. Dos Santos S. N. Pereira T. C. Cabanel M. Chammas R. de Oliveira F. L. (2019). Galectin-3 regulates the expression of tumor glycosaminoglycans and increases the metastatic potential of breast cancer. *J. Oncol.* 2019:9827147. 10.1155/2019/9827147 31949431 PMC6942910

[B48] Phukan R. K. Borkakoty B. J. Phukan S. K. Bhandari K. Mahanta J. Tawsik S. (2018). Association of processed food, synergistic effect of alcohol and HBV with Hepatocellular Carcinoma in a high incidence region of India. *Cancer Epidemiol.* 53 35–41. 10.1016/j.canep.2018.01.005 29360624

[B49] Pirro M. Bianconi V. Paciullo F. Mannarino M. R. Bagaglia F. Sahebkar A. (2017). Lipoprotein(a) and inflammation: a dangerous duet leading to endothelial loss of integrity. *Pharmacol. Res.* 119 178–187. 10.1016/j.phrs.2017.02.001 28185944

[B50] Roessler S. Jia H. L. Budhu A. Forgues M. Ye Q. H. Lee J. S. (2010). A unique metastasis gene signature enables prediction of tumor relapse in early-stage hepatocellular carcinoma patients. *Cancer Res.* 70 10202–10212. 10.1158/0008-5472.Can-10-2607 21159642 PMC3064515

[B51] Rowe G. C. Raghuram S. Jang C. Nagy J. A. Patten I. S. Goyal A. (2014). PGC-1alpha induces SPP1 to activate macrophages and orchestrate functional angiogenesis in skeletal muscle. *Circ. Res.* 115 504–517. 10.1161/circresaha.115.303829 25009290 PMC4524357

[B52] Ruvolo P. P. (2016). Galectin 3 as a guardian of the tumor microenvironment. *Biochim. Biophys. Acta* 1863 427–437. 10.1016/j.bbamcr.2015.08.008 26264495

[B53] Schulze K. Imbeaud S. Letouzé E. Alexandrov L. B. Calderaro J. Rebouissou S. (2015). Exome sequencing of hepatocellular carcinomas identifies new mutational signatures and potential therapeutic targets. *Nat. Genet.* 47:505. 10.1038/ng.3252 25822088 PMC4587544

[B54] Si M. Lang J. (2018). The roles of metallothioneins in carcinogenesis. *J. Hematol. Oncol.* 11:107. 10.1186/s13045-018-0645-x 30139373 PMC6108115

[B55] Slattery M. L. Mullany L. E. Sakoda L. C. Wolff R. K. Stevens J. R. Samowitz W. S. (2018). The PI3K/AKT signaling pathway: associations of miRNAs with dysregulated gene expression in colorectal cancer. *Mol. Carcinog.* 57 243–261. 10.1002/mc.22752 29068474 PMC5760356

[B56] Song L. Mao J. Zhang J. Ibrahim M. M. Li L. H. Tang J. W. (2014). Annexin A7 and its binding protein galectin-3 influence mouse hepatocellular carcinoma cell line in vitro. *Biomed. Pharmacother.* 68 377–384. 10.1016/j.biopha.2013.10.011 24373698

[B57] Song M. Duan Y. Huang T. Zhan L. (2020). Inter-Edge and cloud conversion accelerated user-generated content for virtual brand community. *EURASIP J. Wirel. Commun. Netw.* 2020:14 10.1186/s13638-019-1635-6

[B58] Song S. Z. Lin S. Liu J. N. Zhang M. B. Du Y. T. Zhang D. D. (2019). Targeting of SPP1 by microRNA-340 inhibits gastric cancer cell epithelial-mesenchymal transition through inhibition of the PI3K/AKT signaling pathway. *J. Cell Physiol.* 234 18587–18601. 10.1002/jcp.28497 30953349

[B59] Song Z. Duan Y. Wan S. Sun X. Zou Q. Gao H. (2018). Processing optimization of typed resources with synchronized storage and computation adaptation in Fog computing. *Wirel. Commun. Mob. Comput.* 2018 1–13. 10.1155/2018/3794175

[B60] Szklarczyk D. Morris J. H. Cook H. Kuhn M. Wyder S. Simonovic M. (2017). The STRING database in 2017: quality-controlled protein-protein association networks, made broadly accessible. *Nucleic Acids Res.* 45 D362–D368. 10.1093/nar/gkw937 27924014 PMC5210637

[B61] Totoki Y. Tatsuno K. Covington K. R. Ueda H. Creighton C. J. Kato M. (2014). Trans-ancestry mutational landscape of hepatocellular carcinoma genomes. *Nat. Genet.* 46:1267. 10.1038/ng.3126 25362482

[B62] Trevino V. Falciani F. Barrera-Saldaña H. A. (2007). DNA microarrays: a powerful genomic tool for biomedical and clinical research. *Mol. Med.* 13 527–541. 10.2119/2006-00107.Trevino 17660860 PMC1933257

[B63] Villanueva A. (2019). Hepatocellular carcinoma. *N. Engl. J. Med.* 380 1450–1462. 10.1056/NEJMra1713263 30970190

[B64] Villanueva A. Alsinet C. Yanger K. Hoshida Y. Zong Y. Toffanin S. (2012). Notch signaling is activated in human hepatocellular carcinoma and induces tumor formation in mice. *Gastroenterology* 143 1660–1669.e1667. 22974708 10.1053/j.gastro.2012.09.002 PMC3505826

[B65] Visvader J. E. (2011). Cells of origin in cancer. *Nature* 469 314–322. 10.1038/nature09781 21248838

[B66] Waghray A. Murali A. R. Menon K. N. (2015). Hepatocellular carcinoma: from diagnosis to treatment. *World J. Hepatol.* 7 1020–1029. 10.4254/wjh.v7.i8.1020 26052391 PMC4450179

[B67] Wang S. S. Chen Y. H. Chen N. Wang L. J. Chen D. X. Weng H. L. (2017). Hydrogen sulfide promotes autophagy of hepatocellular carcinoma cells through the PI3K/Akt/mTOR signaling pathway. *Cell Death Dis.* 8:e2688. 10.1038/cddis.2017.18 28333142 PMC5386547

[B68] Wang Y. A. Sun Y. Palmer J. Solomides C. Huang L.-C. Shyr Y. (2017). IGFBP3 modulates lung tumorigenesis and cell growth through IGF1 signaling. *Mol. Cancer Res.* 15 896–904. 10.1158/1541-7786.MCR-16-0390 28330997

[B69] Woo H. G. Park E. S. Cheon J. H. Kim J. H. Lee J.-S. Park B. J. (2008). Gene expression–based recurrence prediction of hepatitis B virus–related human hepatocellular carcinoma. *Clin. Cancer Res.* 14 2056–2064. 18381945 10.1158/1078-0432.CCR-07-1473

[B70] Wurmbach E. Chen Y. B. Khitrov G. Zhang W. Roayaie S. Schwartz M. (2007). Genome-wide molecular profiles of HCV-induced dysplasia and hepatocellular carcinoma. *Hepatology* 45 938–947. 10.1002/hep.21622 17393520

[B71] Xu M. Liu Z. Wang C. Yao B. Zheng X. (2017). EDG2 enhanced the progression of hepatocellular carcinoma by LPA/PI3K/AKT/mTOR signaling. *Oncotarget* 8 66154–66168. 10.18632/oncotarget.19825 29029500 PMC5630400

[B72] Zhang C. Peng L. Zhang Y. Liu Z. Li W. Chen S. (2017). The identification of key genes and pathways in hepatocellular carcinoma by bioinformatics analysis of high-throughput data. *Med. Oncol.* 34:101. 10.1007/s12032-017-0963-9 28432618 PMC5400790

[B73] Zhang P. F. Li K. S. Shen Y. H. Gao P. T. Dong Z. R. Cai J. B. (2016). Galectin-1 induces hepatocellular carcinoma EMT and sorafenib resistance by activating FAK/PI3K/AKT signaling. *Cell Death Dis.* 7:e2201. 10.1038/cddis.2015.324 27100895 PMC4855644

[B74] Zhang Q. Sun S. Zhu C. Zheng Y. Cai Q. Liang X. (2019). Prediction and analysis of weighted genes in hepatocellular carcinoma using bioinformatics analysis. *Mol. Med. Rep.* 19 2479–2488. 10.3892/mmr.2019.9929 30720105 PMC6423588

[B75] Zhang Y. Du W. Chen Z. Xiang C. (2017). Upregulation of PD-L1 by SPP1 mediates macrophage polarization and facilitates immune escape in lung adenocarcinoma. *Exp. Cell Res.* 359 449–457. 10.1016/j.yexcr.2017.08.028 28830685

[B76] Zucman-Rossi J. Villanueva A. Nault J. C. Llovet J. M. (2015). Genetic landscape and biomarkers of hepatocellular carcinoma. *Gastroenterology* 149 1226–1239.e1224. 10.1053/j.gastro.2015.05 26099527

