# Supplementary figures and images for: Integrated Bioinformatics Analysis Reveals Key Candidate Genes and Pathways Associated With Clinical Outcome in Hepatocellular Carcinoma
Source: Front Genet. 2020 Jul 24;11:814. doi: 10.3389/fgene.2020.00814 (PMC7396661; doi:10.3389/fgene.2020.00814)

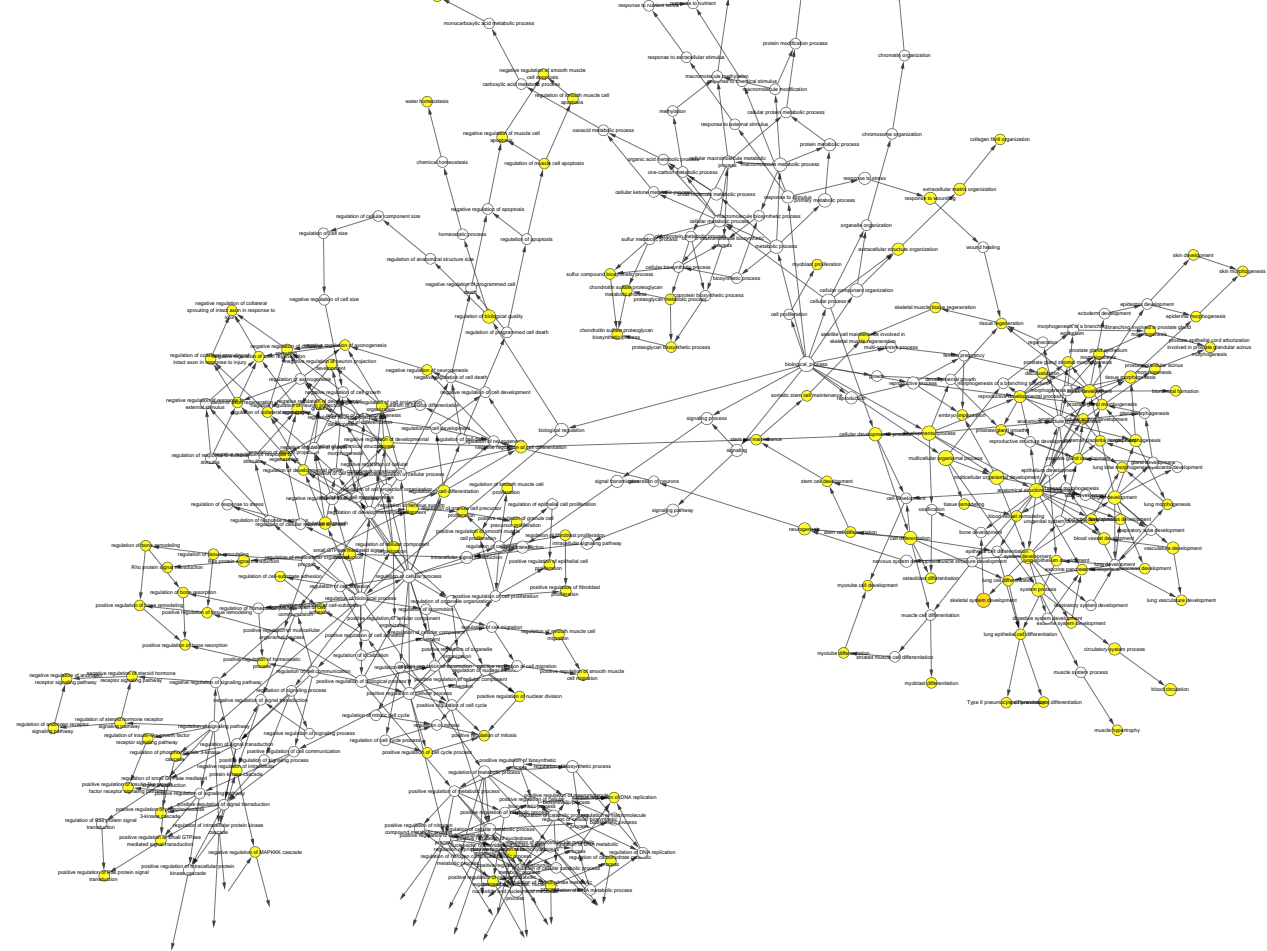

Supplement: FIGURE S1 — The biological process analysis of hub genes using BINGO. [file Data_Sheet_1.PDF]
